# Psychic Muck-Raking

**Published:** 1914-12

**Authors:** 


### Psychic Muck-Raking.

It occurs to us that this term would be a better title for some articles in the current medical literature than Psychic Analysis. For a long time, it has been considered sufficient in discussing the etiology of diabetic exacerbations, for example, to state that the patient had a sudden nerve shock or had suffered a recent bereavement, or that the domestic relations were unfortunate. Why is it necessary to detail the silly, childish, undifferentiated and therefore, more or less homosexual, perverted or incestuous ideas that have obsessed the psychic patient? A recent writer manifests an eagerness to violate the suppositious innocence of the profession regarding homosexuality and points out its many masked forms. For example, a female suffragist, a man who consorts with prostitutes—the homosexuality consisting, as we understand the somewhat recondite reasoning, in the attraction of the masculine aura which hangs about the prostitute from her male visitors—one type of Don Juan, a man who likes the society of other men and, also on account of the femininity displayed, one who prefers the society of women. One of our contemporaries points out that a youth of either sex who is attracted to the opposite sex, is suspected of immorality; if he or she associates with persons of the same sex, of homosexuality; and if, to avoid such suspicions, he or she chooses a solitary life, there is the obvious impugnment of motives. We do not wish to seem prudish to the extent of discouraging the free discussion of any medical topic, organic or functional but when, after reading what purports to be a scientific or clinical article one feels like imploring a friend to tell a smutty story to purify the mental atmosphere, it is time to call for editorial censorship.

